# Leukocyte Chemotactic Factor 2 (LECT2) Amyloidosis Masquerading as Diabetic Kidney Disease in a Hispanic Patient: A Case Report

**DOI:** 10.7759/cureus.111059

**Published:** 2026-06-17

**Authors:** Hema C Aremanda, Ribhi Barta, Emily Chan, Nirupama Vemuri

**Affiliations:** 1 Internal Medicine, Sierra View Medical Center, Porterville, USA; 2 Nephrology, Sierra View Medical Center, Porterville, USA

**Keywords:** amyloid kidney disease, amyloid typing, chronic kidney disease, chronic kidney disease progression, diabetic nephropathy mimic, hispanic ethnicity, lect2 amyloidosis, proteinuria, renal amyloidosis, renal biopsy

## Abstract

Leukocyte chemotactic factor 2 (LECT2) amyloidosis (ALECT2) is a form of renal amyloid deposition that can present with features resembling diabetic kidney disease. We report the case of a woman with long-standing type 2 diabetes mellitus and hypertension who developed progressive renal dysfunction over three years, with her estimated glomerular filtration rate declining from approximately 28 to 10 mL/min/1.73 m² despite stable glycemic control and no identifiable nephrotoxic exposures. Laboratory evaluation revealed minimal albuminuria and unremarkable monoclonal studies, including serum protein electrophoresis, urine protein electrophoresis, and serum free light chain testing. Given the discordance between the degree of proteinuria and the severity of renal function decline, a computed tomography-guided renal biopsy was performed. Histopathologic examination demonstrated Congo red-positive deposits with apple-green birefringence, and immunohistochemical staining confirmed ALECT2. No evidence of systemic amyloid involvement was identified. The patient’s kidney function continued to decline, ultimately progressing to stage 5 chronic kidney disease, and she elected to pursue conservative management. This case illustrates the importance of tissue diagnosis in patients with atypical patterns of chronic kidney disease.

## Introduction

Amyloidosis comprises a heterogeneous group of disorders characterized by extracellular deposition of misfolded fibrillar proteins, leading to progressive organ dysfunction [1,2]. More than 30 amyloidogenic precursor proteins have been identified, with renal involvement being one of the most common manifestations [1,3]. Accurate amyloid typing is essential because prognosis and treatment vary substantially among subtypes [1,2].

Leukocyte chemotactic factor 2 (LECT2) amyloidosis (ALECT2) is a recently recognized form of amyloid disease that predominantly affects the kidneys and occurs most frequently in individuals of Hispanic, Middle Eastern, and South Asian ancestry [4-6]. Unlike immunoglobulin light-chain (AL) or serum amyloid A (AA) amyloidosis, ALECT2 is generally confined to the kidneys and is not associated with plasma cell dyscrasias or chronic inflammatory disorders [4,5]. Patients typically present with slowly progressive chronic kidney disease, bland urinary sediment, and absent or mild proteinuria, often without nephrotic syndrome or systemic organ involvement [4,6-8]. Consequently, ALECT2 is frequently misdiagnosed as diabetic or hypertensive kidney disease, particularly in patients with long-standing metabolic comorbidities [4,7].

Definitive diagnosis requires renal biopsy with Congo red staining to confirm amyloid deposition, followed by accurate amyloid typing [3,4]. Renal involvement characteristically demonstrates predominant cortical interstitial amyloid deposition, with relatively limited glomerular involvement, a pattern that differs from the more extensive glomerular deposition commonly observed in AL amyloidosis. Because histopathologic appearance alone cannot reliably establish the amyloid subtype, immunohistochemistry and laser microdissection with mass spectrometry-based proteomic analysis are valuable diagnostic tools, with the latter considered the reference standard when available [3]. Accurate classification is critical to avoid inappropriate therapy, including unnecessary chemotherapy intended for AL amyloidosis [2-4].

Recognition of ALECT2 has important clinical implications. Although no disease-modifying therapy currently exists, accurate diagnosis facilitates prognostic counseling, avoidance of harmful treatments, informed transplant planning, and optimization of conservative kidney management [4-6,9]. Diagnosis in routine clinical practice remains challenging because affected patients frequently have common metabolic comorbidities that provide an alternative explanation for chronic kidney disease.

We report a patient with long-standing diabetes mellitus and hypertension whose progressive kidney dysfunction was initially attributed to diabetic kidney disease but was ultimately found to represent ALECT2. This case highlights the diagnostic challenges posed by this underrecognized entity and underscores the importance of considering renal biopsy when kidney function declines disproportionately to the degree of albuminuria in patients with presumed diabetic kidney disease.

## Case presentation

Hispanic female in her late sixties with a medical history significant for type 2 diabetes mellitus for approximately 20 years, hypertension for approximately 15 years, hyperlipidemia, and hypothyroidism was followed in the nephrology clinic for a progressive decline in kidney function. Glycemic control remained stable during follow-up, with hemoglobin A1c values ranging from 6.1% to 6.4%, and blood pressure was generally well controlled during outpatient visits, typically measuring below 140/90 mmHg. Her medication regimen included insulin therapy, amlodipine for hypertension, levothyroxine, and statin therapy. She denied the use of nonsteroidal anti-inflammatory drugs, herbal supplements, over-the-counter nephrotoxic medications, intravenous contrast exposure, or other known nephrotoxic agents. She lived independently and reported no tobacco, alcohol, or illicit drug use.

Initial evaluation

The patient initially presented with nonspecific constitutional symptoms, including nausea, reduced appetite, mild unintentional weight loss, and intermittent abdominal discomfort. Vital signs were stable at presentation. Physical examination was unremarkable, with no peripheral edema, rash, or other overt features of systemic amyloidosis. There was no documented diabetic retinopathy, peripheral neuropathy, or history of diabetic foot disease.

Initial laboratory evaluation revealed impaired kidney function with mildly elevated creatinine and preserved serum albumin levels. Urinalysis demonstrated low-grade proteinuria without hematuria. Glycemic control was stable throughout this period. These findings are summarized in Table 1.

**Table 1:** Initial laboratory evaluation.

Parameter	Value	Units	Reference range
Serum creatinine	2.5-2.8	mg/dL	0.6-1.2
Estimated GFR	~28	mL/min/1.73 m²	>60
Blood urea nitrogen	28-31	mg/dL	7-20
Serum potassium	4.3-4.5	mmol/L	3.5-5.1
Serum albumin	3.8	g/dL	3.5-5.0
Hemoglobin A1c	6.1-6.4	%	5.7-6.4
Urinalysis	1+ protein	-	Negative
Hematuria	Absent	-	Negative
Urine microalbumin/creatinine ratio	22–34	mg/g	<30

Serologic evaluation for monoclonal gammopathy, including serum protein electrophoresis, urine protein electrophoresis, and serum free light chain analysis, was unremarkable.

Worsening kidney function

Despite continued blood pressure control and stable glycemic management, the patient demonstrated a progressive decline in renal function over subsequent follow-up visits. Serial laboratory measurements revealed a gradual increase in serum creatinine accompanied by a steady reduction in estimated glomerular filtration rate over a 36-month period. Blood urea nitrogen levels increased concurrently, and intermittent mild hyperkalemia was observed. Proteinuria remained minimal throughout the disease course, and serum albumin levels remained within normal limits. Longitudinal renal function trends are summarized in Table 2.

**Table 2:** Longitudinal trends in renal function over 36 months. GFR, glomerular filtration rate

Parameter	Baseline (Month 0)	6 months	12 months	24 months	36 months	Units	Reference range
Serum creatinine	2.6	2.9	3.3	3.7	4.4	mg/dL	0.6-1.2
Estimated GFR	~28	~24	~20	~14	~10	mL/min/1.73 m²	≥60
Blood urea nitrogen	36	42	50	56	62	mg/dL	7-20
Serum potassium	4.6	4.9	5.1	5.3	5.5	mmol/L	3.5-5.1

This discordance between progressive eGFR decline and minimal albuminuria, together with preserved serum albumin, bland urinary sediment, absent diabetic retinopathy, and negative monoclonal studies, prompted renal biopsy.

Renal biopsy

**Figure 1:**
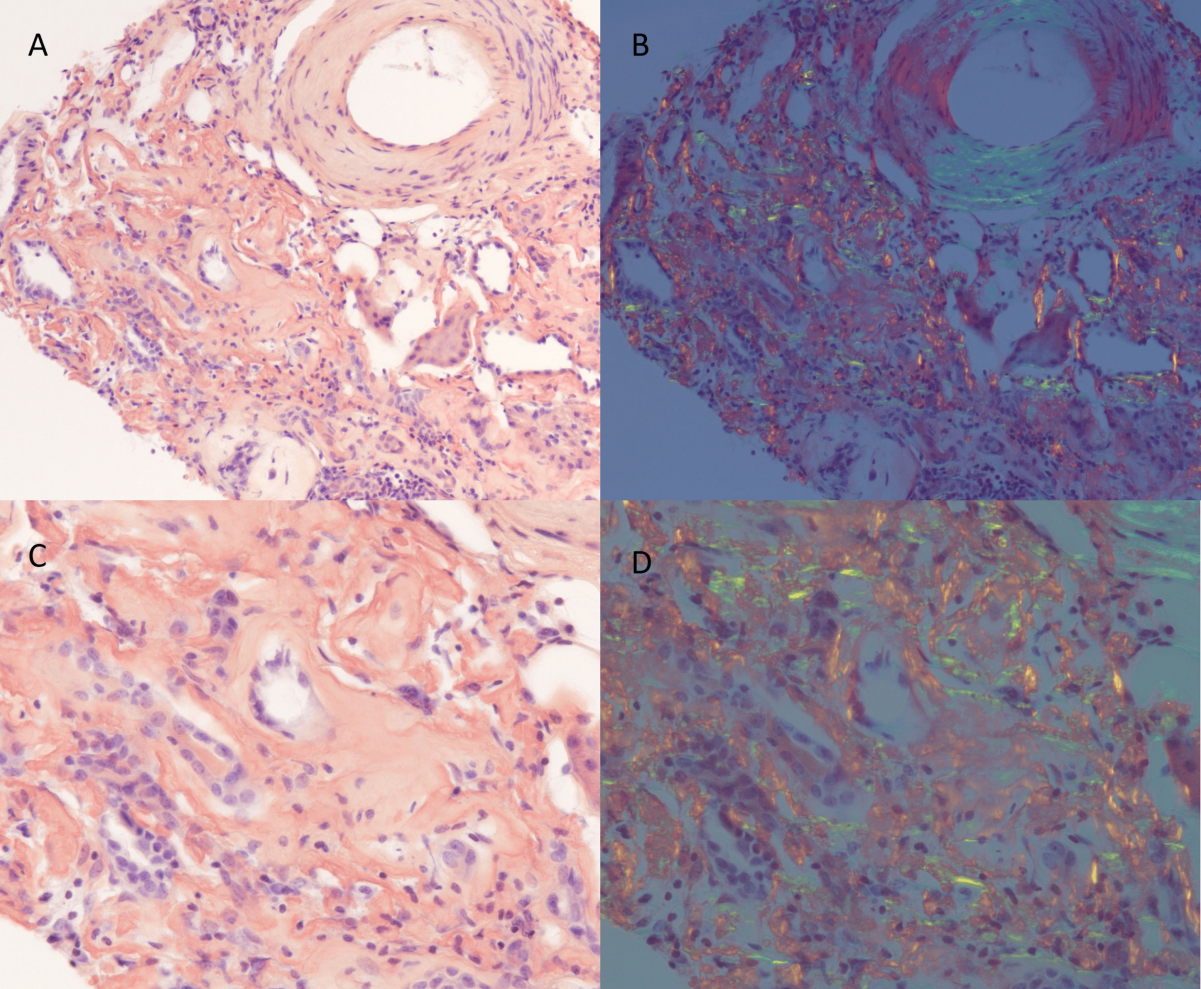
Congo red staining under bright-field and polarized microscopy. (A) Low-power Congo red stain demonstrating predominantly cortical interstitial amyloid deposition. (B) Low-power polarized microscopy demonstrating characteristic apple-green birefringence of the amyloid deposits. (C) High-power Congo red stain showing amorphous extracellular amyloid deposits. (D) High-power polarized microscopy confirming apple-green birefringence of the deposits.

Given the discordance between mild proteinuria and accelerated loss of kidney function, a computed tomography-guided biopsy of the left kidney was performed. Histopathologic examination demonstrated Congo red-positive amyloid deposition with characteristic apple-green birefringence under polarized microscopy, confirming amyloid deposition. Low-power examination demonstrated predominantly cortical interstitial amyloid deposition, whereas high-power examination highlighted amorphous extracellular deposits with relatively limited glomerular involvement (Figure 1).

Light microscopy identified three glomeruli, one of which was globally sclerosed. The remaining glomeruli showed no segmental sclerosis, crescents, or endocapillary hypercellularity. Mesangial areas were unremarkable, and there was no evidence of diabetic glomerulosclerosis. Approximately 20% to 30% tubular atrophy and interstitial fibrosis were present. Amyloid deposition was predominantly cortical and interstitial in distribution, involving nearly 50% of the cortical tissue as Periodic acid-Schiff (PAS)-negative amorphous extracellular deposits with relatively limited glomerular involvement.

**Figure 2:**
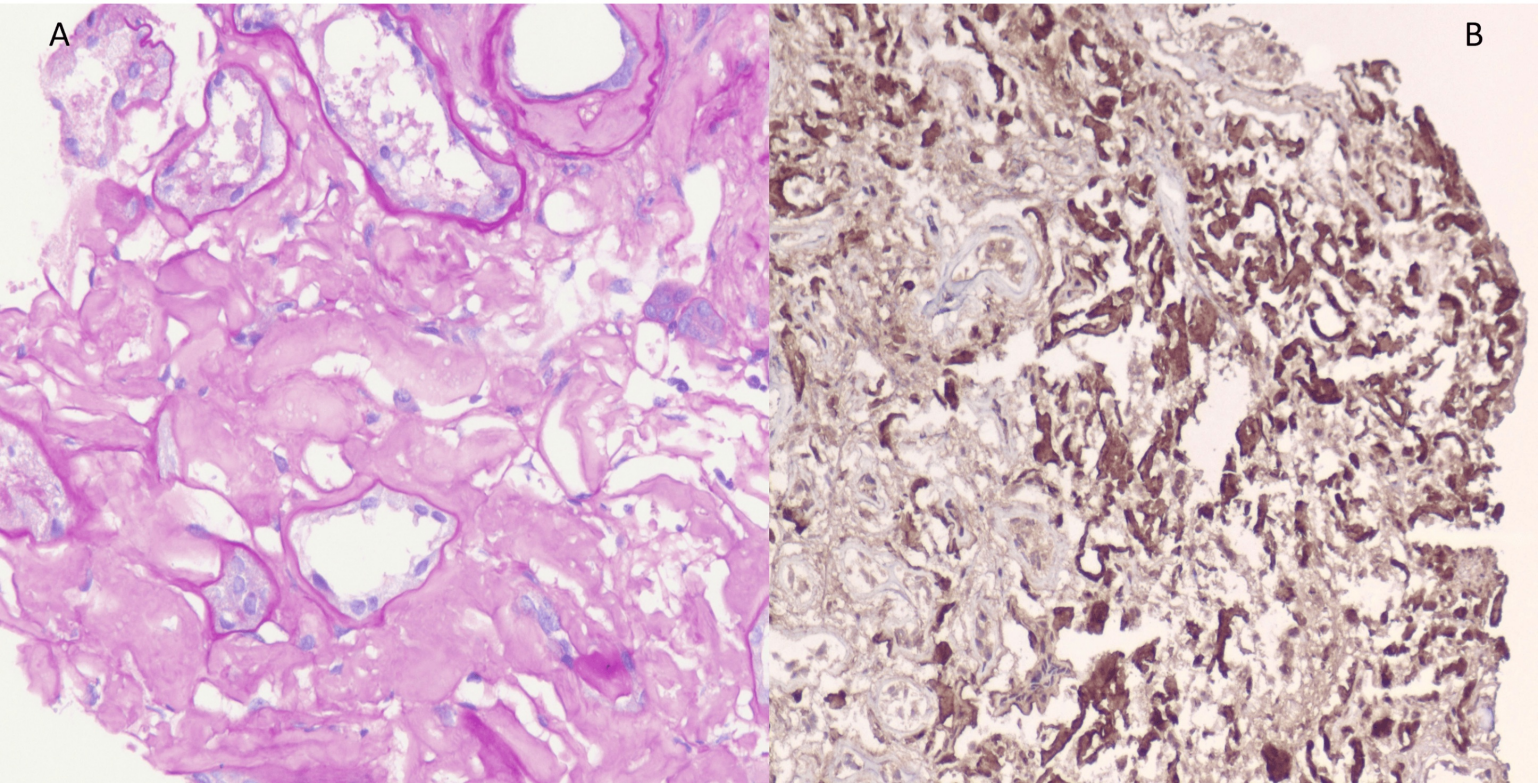
Periodic acid-Schiff (PAS) stain and leukocyte chemotactic factor 2 (LECT2) immunohistochemistry. (A) PAS stain demonstrating PAS-negative amorphous extracellular deposits predominantly involving the cortical interstitium. (B) LECT2 immunohistochemistry demonstrating strong positive staining of the amyloid deposits, supporting the diagnosis of ALECT2.

Representative PAS staining demonstrated PAS-negative extracellular deposits, while immunohistochemistry showed strong positivity for LECT2. Stains for immunoglobulin light chains, serum amyloid A, and transthyretin were negative, supporting the diagnosis of ALECT2 (Figure 2). Laser microdissection with mass spectrometry was not performed because it was not available as part of the diagnostic workup at the treating institution.

**Figure 3:**
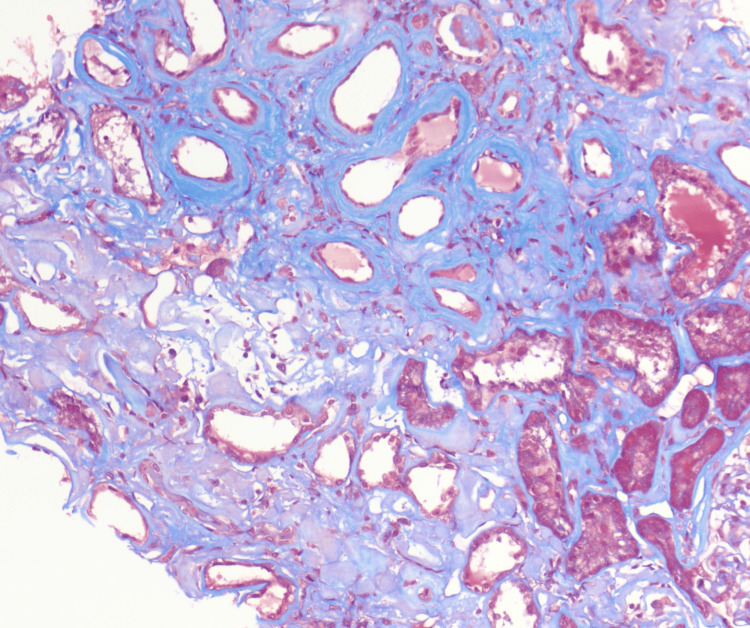
Masson's trichrome stain demonstrating chronic tubulointerstitial scarring and interstitial expansion associated with renal amyloid deposition.

Masson's trichrome stain highlighted chronic tubulointerstitial injury with approximately 20% to 30% tubular atrophy and interstitial fibrosis, supporting the chronicity of the underlying renal injury and the characteristic interstitial-predominant pattern of ALECT2 (Figure 3).

**Figure 4:**
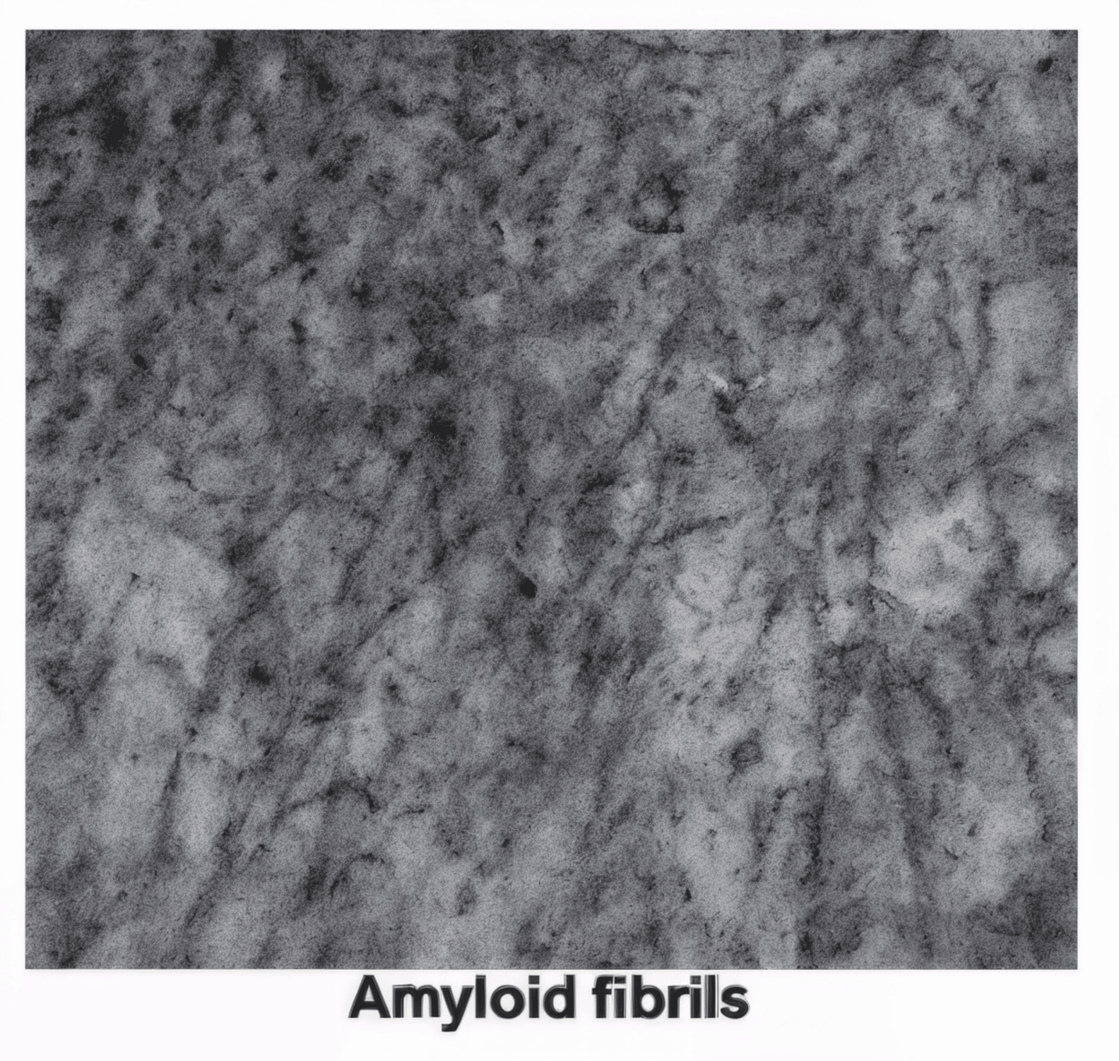
Transmission electron microscopy demonstrating randomly arranged, nonbranching fibrils characteristic of amyloid deposition.

Transmission electron microscopy revealed randomly arranged, nonbranching fibrils measuring approximately 10 nm in diameter, consistent with amyloid deposition (Figure 4). Ultrastructural examination demonstrated extensive interstitial fibril deposition with segmental mesangial involvement and relative preservation of the glomerular basement membranes. No electron-dense immune complex or powdery deposits were identified. These findings were consistent with the characteristic interstitial-predominant pattern described in ALECT2 and indicated that ALECT2, rather than diabetic glomerulosclerosis, was the dominant pathologic process underlying the patient's renal decline.

Subsequent clinical course

Following the diagnosis, renal function continued to deteriorate, ultimately progressing to stage 5 chronic kidney disease. The patient developed complications of advanced kidney disease, including recurrent hyperkalemia managed with patiromer, secondary hyperparathyroidism treated with calcitriol and vitamin D supplementation, and chronic fatigue.

Evaluation for extrarenal amyloid involvement did not demonstrate systemic disease. The patient had no clinical evidence of peripheral or autonomic neuropathy, macroglossia, or nephrotic syndrome. Previously obtained monoclonal studies were unremarkable. Transthoracic echocardiography demonstrated no evidence of infiltrative cardiomyopathy, computed tomography of the abdomen showed no hepatosplenomegaly, and colonoscopy revealed ischemic colitis without evidence of gastrointestinal amyloid involvement. In the absence of monoclonal gammopathy or systemic features suggestive of AL amyloidosis, bone marrow biopsy was deferred.

After extensive counseling regarding renal replacement therapy, the patient elected to decline dialysis and chose conservative kidney management.

## Discussion

ALECT2 is an increasingly recognized but underdiagnosed cause of renal amyloid deposition, particularly among Hispanic, Middle Eastern, and South Asian populations [4-7]. Unlike immunoglobulin light-chain (AL) or serum amyloid A (AA) amyloidosis, ALECT2 is typically confined to the kidneys and is characterized by slowly progressive renal dysfunction, bland urinary sediment, and absent or mild proteinuria without significant extrarenal involvement [1,2,4-8]. Consequently, affected patients are frequently misdiagnosed with diabetic or hypertensive kidney disease, especially in the presence of common metabolic comorbidities.

In our patient, long-standing diabetes and hypertension initially supported a presumed diagnosis of diabetic kidney disease. However, minimal albuminuria, preserved serum albumin, absence of diabetic retinopathy, and progressive estimated glomerular filtration rate (eGFR) decline out of proportion to the degree of proteinuria represented an important clinicopathologic discordance that prompted renal biopsy. The educational value of this case lies not in an unusual manifestation of ALECT2 but in illustrating how common metabolic conditions may obscure the diagnosis and delay appropriate evaluation.

Accurate amyloid typing is essential because treatment and prognosis differ substantially among amyloid subtypes. Congo red staining alone cannot distinguish amyloid precursors, and laser microdissection with mass spectrometry-based proteomic analysis remains the reference standard for classification when available [3,4]. In the present case, mass spectrometry was unavailable, but the diagnosis was supported by Congo red-positive deposits, characteristic ultrastructural fibrils, positive LECT2 immunohistochemistry, and negative staining for immunoglobulin light chains, serum amyloid A, and transthyretin.

The pathogenesis of ALECT2 remains incompletely understood. LECT2 is a hepatically synthesized cytokine involved in inflammatory signaling and tissue repair, and current evidence suggests that amyloid deposition results from abnormal protein folding rather than overproduction of the precursor protein. The marked renal tropism of ALECT2 remains unexplained, although its characteristic cortical interstitial distribution suggests tissue-specific factors favoring fibril formation. A common LECT2 gene polymorphism, c.172G>A (rs31517), has been associated with populations at increased risk, including Hispanics, although its precise pathogenic role remains uncertain [4,7]. Circulating and urinary LECT2 assays are under investigation as potential noninvasive biomarkers but are not currently used in routine clinical practice.

The histopathologic findings in this case closely reflect the characteristic renal pattern of ALECT2. Biopsy demonstrated predominantly interstitial amyloid deposition with relatively limited glomerular involvement, moderate tubular atrophy and interstitial fibrosis, significant global glomerulosclerosis, and no evidence of diabetic glomerulosclerosis. Electron microscopy showed randomly arranged nonbranching fibrils with extensive interstitial deposition and relative preservation of the glomerular basement membranes. These findings support the interstitial-predominant pattern reported in ALECT2 and likely contributed to progressive renal dysfunction despite minimal proteinuria.

Cardiac, neurologic, and gastrointestinal involvement are uncommon, and ALECT2 is generally kidney-limited, contributing to relatively favorable overall survival compared with systemic amyloidoses, although progression to kidney failure is common once chronic tubulointerstitial injury develops [4-6,8]. Published series suggest an average eGFR decline of approximately 4 mL/min/1.73 m² per year [8]. Our patient experienced a more rapid decline of approximately 6 mL/min/1.73 m² per year. Although diabetes and hypertension may have contributed to the overall burden of chronic kidney disease, the absence of diabetic glomerulosclerosis and the extensive interstitial amyloid deposition suggest that ALECT2 was the principal pathologic process underlying disease progression.

No disease-modifying therapy currently exists for ALECT2, and management focuses on supportive care and treatment of chronic kidney disease complications [1,4,8]. Kidney transplantation is a reasonable option for eligible patients with end-stage kidney disease, with generally favorable graft outcomes despite occasional histologic recurrence of amyloid deposits [4,8]. In the present case, the patient elected conservative kidney management and declined dialysis and kidney transplantation; consequently, transplant evaluation was not pursued.

This case highlights the importance of maintaining diagnostic vigilance when evaluating chronic kidney disease with an atypical clinical course. In patients with minimal proteinuria, preserved serum albumin, and unexpectedly rapid eGFR decline, particularly among high-risk ethnic populations, renal biopsy remains a critical diagnostic tool. Early recognition of ALECT2 facilitates accurate prognostication, avoids inappropriate therapy, and supports informed long-term management planning.

## Conclusions

ALECT2 is an underrecognized cause of chronic kidney disease, particularly among Hispanic patients, and may clinically resemble diabetic or hypertensive nephropathy due to its presentation with mild proteinuria, progressive renal dysfunction, and normal monoclonal protein studies. This case illustrates how reliance on clinical history alone may delay accurate diagnosis in selected patients with long-standing metabolic comorbidities whose renal decline appears disproportionate to the degree of albuminuria.

Renal biopsy should be considered when chronic kidney disease follows an atypical clinical trajectory, particularly when there is progressive eGFR decline despite minimal proteinuria and preserved serum albumin. In this case, histopathologic evaluation demonstrated an interstitial-predominant amyloid deposition pattern without evidence of diabetic glomerulosclerosis, highlighting the value of tissue diagnosis in avoiding diagnostic anchoring. Accurate amyloid typing is critical because treatment and prognosis differ substantially among amyloid subtypes; laser microdissection with mass spectrometry remains the preferred method for definitive classification when available.

Recognition of ALECT2 may be particularly relevant in high-risk populations, including Hispanic patients, in whom the LECT2 c.172G>A (rs31517) polymorphism has been reported more frequently, although its precise pathogenic significance remains incompletely understood. Clinicians should maintain suspicion for ALECT2 in selected patients with unexplained eGFR decline, preserved serum albumin, and minimal proteinuria. Earlier recognition may help avoid inappropriate therapy, support accurate prognostic counseling, and guide individualized discussions regarding conservative kidney management, dialysis, or transplantation.
